# Ganciclovir‐induced drug reaction with eosinophilia and systemic symptoms

**DOI:** 10.1002/ski2.269

**Published:** 2023-07-13

**Authors:** Shohei Kitayama, Teruhiko Makino, Kuniko Fujita, Shuichi Mori, Fumina Furukawa, Ko Kagoyama, Megumi Mizawa, Tadamichi Shimizu

**Affiliations:** ^1^ Faculty of Medicine Department of Dermatology Academic Assembly University of Toyama Toyama Japan

## Abstract

Drug reaction with eosinophilia and systemic symptoms (DRESS) is a severe cutaneous adverse reaction involving multiorgan failure, with a complex interaction of various drugs, human herpesvirus reactivation and immune abnormalities suggested as the aetiology. We herein present the case of a 70‐year‐old man with a one‐week history of fever, facial oedema, erythematous macules and purpura on his trunk and extremities. He had anti‐TIF1γ antibody‐positive dermatomyositis and was treated with prednisolone sodium succinate (20 mg/day). Three weeks earlier, he was treated with ganciclovir (250 mg/day) for 7 days to treat asymptomatic cytomegalovirus viraemia. Laboratory investigations revealed eosinophilia with atypical lymphocytes and elevated liver enzyme levels. A histological examination showed interface dermatitis with necrotic keratinocytes, perivascular infiltration of lymphocytes and eosinophils in the upper dermis and erythrocyte extravasation without vasculitis. A lymphocyte transformation test (LTT) was positive for ganciclovir (stimulation index: 260%; normal: <180%). We diagnosed DRESS caused by ganciclovir on the basis of clinical findings and course (Definite; RegiSCAR score: 7). He was treated with prednisolone sodium succinate (40 mg/day) and topical clobetasol propionate (0.05%) ointment twice daily. After the initiation of treatment, the skin lesions and laboratory abnormalities gradually improved. To our knowledge, this is the first case of DRESS caused by ganciclovir. The patients in whom ganciclovir is used are often immunosuppressed and may be overlooked as the causative drug for DRESS by conventional skin tests. We considered that LTT is useful for identifying causative drugs of DRESS, especially in immunosuppressed patients, such as the present case.

## Graphical abstract

This is the first case of ganciclovir‐induced drug reaction with eosinophilia and systemic symptoms. The drug is often used in immunosuppressed patients, and adverse cutaneous reactions may be missed due to difficulties with allergological work‐up.

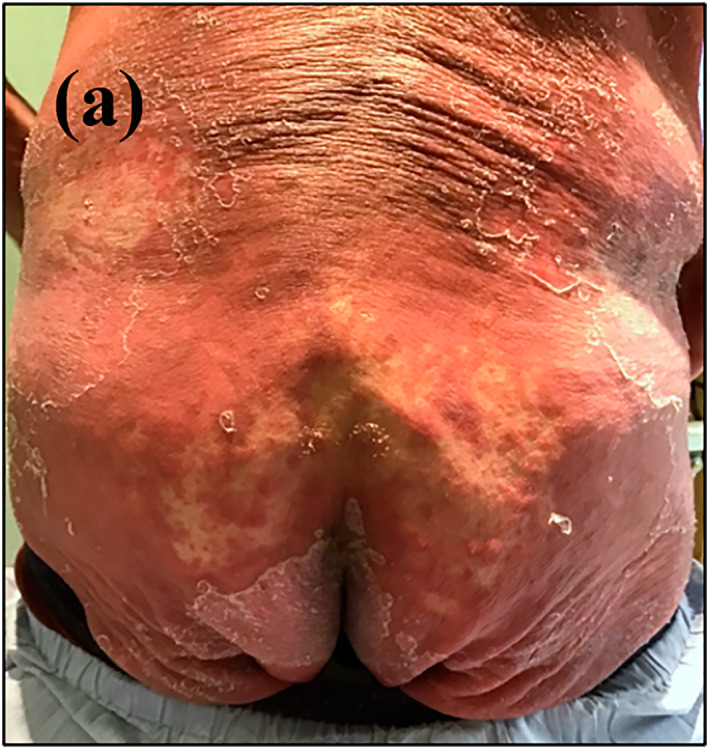

## 1 CASE

Drug reaction with eosinophilia and systemic symptoms (DRESS) is a severe cutaneous adverse reaction (SCAR) involving multiorgan failure, with a complex interaction of various drugs, human herpesvirus reactivation and immune abnormalities suggested as the aetiology. Patch tests and delayed intradermal testing are common methods to identify the causative drug of DRESS, but its identification is often difficult in conditions of high disease activity or during immunosuppressive therapy.^1^ We herein report a case of DRESS in which the causative drug was identified as ganciclovir using the lymphocyte transformation test (LTT).

**FIGURE 1:**
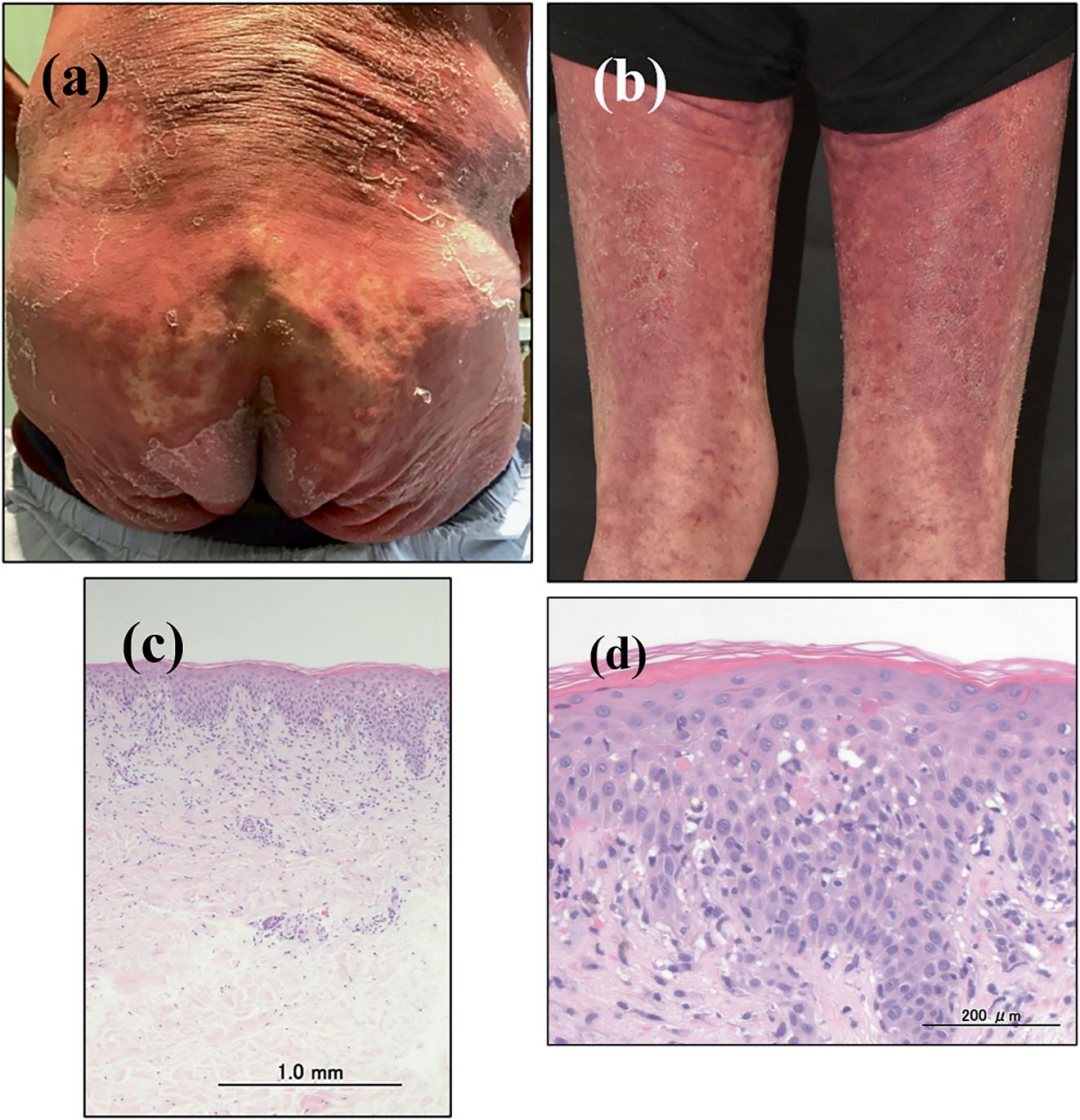
(a, b) The clinical findings of the patient at the initial presentation. A physical examination revealed maculopapular exanthema with scaling on the hip (a) and purpura on the lower extremities (b). (c, d) A histopathological examination of the rash revealed interface dermatitis with vacuolar change, colloid bodies and sparse inflammation. Perivascular infiltration of lymphocytes and eosinophils in the upper dermis and erythrocyte extravasation without vasculitis were also observed (haematoxylin‐eosin staining).

A 70‐year‐old man was evaluated for cutaneous rash and fever. He had anti‐TIF1γ antibody‐positive dermatomyositis and was treated with prednisolone sodium succinate (sPSL [20 mg/day, intravenously]). The disease activity was well controlled, and the only other drugs used were pentamidine isetionate and lansoprazole, which have been used for more than 3 months. Three weeks earlier, he was treated with ganciclovir (250 mg/day) for 7 days to treat asymptomatic cytomegalovirus viraemia diagnosed by a weekly cytomegalovirus antigenemia test (C7‐HRP). One week earlier, an itchy rash developed and spread over his trunk (Figure 1a). Furthermore, purpura on the lower extremities (Figure 1b) and facial oedema developed. Fever (39.3°C) with lymphadenopathy (axilla and inguina) was observed. Mucosal involvement and muscle weakness were not observed. Laboratory investigations revealed a leucocyte count of 3910/mm^3^ (eosinophils: 12.5%) with atypical lymphocytes and elevated transaminase levels (aspartate aminotransferase, 144 U/L [normal 13–30 U/L], alanine aminotransferase, 433 U/L [normal 10–42 U/L]). His serum creatinine and creatinine kinase levels were within the normal ranges. Tests for herpes simplex virus, varicella‐zoster virus and Epstein‒Barr virus were consistent with past infection. The patient's blood was negative for HHV‐6 DNA and C7‐HRP‐positive cells. In addition, antinuclear antibodies, pharyngeal culture, blood cultures, PCR for COVID‐19 and serological tests for hepatitis B virus/hepatitis C virus and mycoplasma were all negative. A histological examination showed vacuolar interface with colloid bodies and sparse inflammation. Additionally, perivascular infiltration of lymphocytes and eosinophils in the upper dermis and erythrocyte extravasation without vasculitis were observed (Figure 1c,d). An LTT was positive for ganciclovir (stimulation index: 260%; normal: <180%) and negative for pentamidine isetionate and lansoprazole. The clinical findings and course indicated ganciclovir‐induced DRESS (Definite; RegiSCAR score: 7 [enlarged lymph nodes in >2 different anatomical areas, eosinophil ratio, presence of atypical lymphocytes, skin rash extent >50% body surface area and suggesting DRESS, liver involvement, and other potential causes ruled out]).^1^ He was treated with sPSL (40 mg/day) and topical clobetasol propionate (0.05%) ointment twice daily. After the initiation of treatment, the skin lesions and laboratory abnormalities gradually improved. The patient is now being carefully observed with the administration of sPSL (20 mg/day). In addition, weekly C7‐HRP was measured from the end of ganciclovir treatment and throughout the treatment period of DRESS, with all results found to be negative.

DRESS is a delayed‐type drug hypersensitivity reaction and often develops within 1 week to 3 months after the use of the responsible drug. The latency period varies depending on the responsible drug and is known to be longer for antiepileptic drugs than for antimicrobial drugs.^2^ The course of the disease is mainly divided into two phases: an early phase within approximately 1–2 months after onset and a later phase.^1^ In the early phase, DRESS symptoms, including fever, liver injury, and haematological abnormalities, are followed by reactivation of herpesviruses (especially cytomegalovirus). The later phase is characterised by flare‐ups of DRESS symptoms and the development of autoimmune diseases. The mortality rate for DRESS is estimated to be between 2% and 10%, depending on age, race and comorbidities. It has been suggested that the duration of exposure to the causative drug after the onset of DRESS is associated with its severity and the incidence of complications.^3^ Therefore, it is important to be aware of even drugs that infrequently cause DRESS.

The differential diagnosis of DRESS includes autoimmune diseases and infectious diseases.^1^ In the present case, it was particularly important to distinguish dermatomyositis and CMV infection. Haematological abnormalities (eosinophilia and the presence of atypical lymphocytes) associated with elevated transaminases and eosinophil infiltration of the skin are atypical for dermatomyositis. In addition, we also ruled out the possibility of persistent CMV reactivation after treatment with ganciclovir. This is because C7‐HRP is negative at all points during DRESS treatment, and intensified systemic glucocorticoid therapy tended to improve the clinical course of the symptoms without antiviral therapy.

DRESS has been reported to be caused by a limited number of drugs, with vancomycin, trimethoprim‐sulfamethoxazole, allopurinol, lamotrigine and carbamazepine accounting for nearly half of all causative drugs in the United States.^1^ Ganciclovir is widely used to treat cytomegalovirus infections, but SCAR is rare. To our knowledge, this is the first reported case of DRESS caused by ganciclovir. The precise mechanism by which ganciclovir causes DRESS is unclear. It is known that a population that is hypersensitive to one drug tends to be hypersensitive to other drugs with similar chemical structures. Recently, certain HLAs were reported to be associated with pharmacogenetic susceptibility to specific drug‐induced DRESS, indicating the existence of certain immunogenetically susceptible populations.^1^ Thus, in patients with certain HLAs, specific chemical structures might be recognised by the immune system and trigger the onset of DRESS. In fact, 2 of 15 patients with specific HLA‐class II haplotype‐restricted vancomycin‐induced DRESS showed cross‐reactivity to teicoplanin and telavancin, which share the core chemical structure of vancomycin.^4^ One drug that shares the chemical structure of a 2‐aminopurine nucleus with ganciclovir is the anti‐herpesvirus drug valacyclovir, and four cases of DRESS due to this drug have been reported.^5^, ^6^ Vernassiere et al. also suggested that ganciclovir and valacyclovir may represent a common drug allergy because of their shared chemical structure.^7^ We hypothesised that ganciclovir may cause DRESS through a similar pathomechanism to valacyclovir‐induced DRESS in a population immunogenetically susceptible to its chemical structure, although further analyses will be required to prove this hypothesis.

An LTT was used to identify ganciclovir as a causative drug. To identify the causative drug, it is necessary to investigate the patient's drug history (prior probability estimation), as in this case, and the method of identification. Regarding the allergological work‐up, drugs such as ganciclovir may be overlooked in patients taking various drugs because of their low imputability.^6^ Ganciclovir is often used for patients in highly immunosuppressed states (e.g., after haematopoietic stem cell transplantation or during immunosuppressive therapy). In these situations, a patch test and delayed intradermal testing, a common method for identifying the cause of SCARs, may yield false‐negative results.^1^, ^8^ This may contribute to the rarity of reported cases of ganciclovir‐induced SCAR. Furthermore, as this is an in vivo test, it may cause DRESS symptoms to flare‐up. On the other hand, LTT is an in vitro test that detects the antigen‐stimulated proliferative potential of drug‐specific memory T cells. Its sensitivity and specificity are reportedly superior in comparison to skin tests for identifying the causative drug of DRESS.^9^ Furthermore, it has been reported that the results of LTT are not influenced by immunosuppressive therapy in cases of DRESS.^10^ Although LTT inspection fees are relatively high and available medical facilities may be limited, we considered that LTT is useful for identifying causative drugs of DRESS, especially in immunosuppressed patients, such as the present case.

## CONFLICT OF INTEREST STATEMENT

None to declare.

## AUTHOR CONTRIBUTIONS

**Shohei Kitayama**: Conceptualization (lead); data curation (lead); formal analysis (lead); investigation (lead); methodology (equal); writing – original draft (lead); writing – review & editing (equal). **Teruhiko Makino**: Investigation (equal); methodology (equal); supervision (equal); writing – original draft (equal); writing – review & editing (equal). **Kuniko Fujita**: Investigation (supporting); methodology (supporting). **Shuichi Mori**: Investigation (supporting); methodology (supporting). **Fumina Furukawa**: Investigation (supporting); methodology (supporting). **Ko Kagoyama**: Investigation (supporting); methodology (supporting). **Megumi Mizawa**: Investigation (equal); methodology (equal); writing – original draft (equal); writing – review & editing (equal). **Tadamichi Shimizu**: Funding acquisition (equal); investigation (equal); methodology (equal); resources (equal); writing – original draft (equal); writing – review & editing (equal).

## ETHICS STATEMENT

In Japan, IRB review is not normally undertaken for single case reports. Our institutions cope with this in the same way. If it is required for submission to your journal, it will be reviewed by the IRB.

## Data Availability

The data that support the findings of this study are available on request to the corresponding author. The data are not publicly available due to patient's privacy reasons.
